# Cardiac Fibroblasts Aggravate Viral Myocarditis: Cell Specific Coxsackievirus B3 Replication

**DOI:** 10.1155/2014/519528

**Published:** 2014-10-13

**Authors:** Diana Lindner, Jia Li, Konstantinos Savvatis, Karin Klingel, Stefan Blankenberg, Carsten Tschöpe, Dirk Westermann

**Affiliations:** ^1^Clinic for General and Interventional Cardiology, University Heart Center Hamburg, Martinistraße 52, 20246 Hamburg, Germany; ^2^German Center for Cardiovascular Research (DZHK), Partner Sites, Hamburg/Kiel/Lübeck, Germany; ^3^Department of Cardiology and Pneumology, Charité-Universitätsmedizin Berlin, Campus Benjamin Franklin (CBF), Berlin, Germany; ^4^Department of Molecular Pathology, Institute for Pathology, Eberhard Karls University of Tübingen, Tübingen, Germany; ^5^German Center for Cardiovascular Research (DZHK), Partner Sites, Berlin, Germany

## Abstract

Myocarditis is an inflammatory disease caused by viral infection. Different subpopulations of leukocytes enter the cardiac tissue and lead to severe cardiac inflammation associated with myocyte loss and remodeling. Here, we study possible cell sources for viral replication using three compartments of the heart: fibroblasts, cardiomyocytes, and macrophages. We infected C57BL/6j mice with Coxsackievirus B3 (CVB3) and detected increased gene expression of anti-inflammatory and antiviral cytokines in the heart. Subsequently, we infected cardiac fibroblasts, cardiomyocytes, and macrophages with CVB3. Due to viral infection, the expression of TNF-α, IL-6, MCP-1, and IFN-*β* was significantly increased in cardiac fibroblasts compared to cardiomyocytes or macrophages. We found that in addition to cardiomyocytes cardiac fibroblasts were infected by CVB3 and displayed a higher virus replication (132-fold increase) compared to cardiomyocytes (14-fold increase) between 6 and 24 hours after infection. At higher virus concentrations, macrophages are able to reduce the viral copy number. At low virus concentration a persistent virus infection was determined. Therefore, we suggest that cardiac fibroblasts play an important role in the pathology of CVB3-induced myocarditis and are another important contributor of virus replication aggravating myocarditis.

## 1. Introduction

Myocarditis is an inflammatory disease of the heart muscle often resulting in cardiac dysfunction and death, especially in young patients [1, 2]. The gold standard for diagnosing myocarditis is based on histopathology from endomyocardial biopsies, in which clear inflammatory cellular infiltrates are present [1, 3–5].

One of the most common causes for acute myocarditis is a cardiac infection with viruses, especially with enteroviruses of the Coxsackievirus B family [1, 6, 7]. This virus-induced myocarditis remains still one major cause of dilated cardiomyopathy. Virus-mediated cell lysis leads to destruction of cardiac cells, especially cardiomyocytes. The destroyed cardiomyocytes are then replaced by fibrosis which finally may lead to dilated cardiomyopathy.

CVB3-induced viral myocarditis is a well-established model to elucidate viral myocarditis in mice [8–14]. In this model, mice were infected intraperitoneally with CVB3 and within a few days a severe inflammation and virus replication can be observed in the cardiac tissue. In general, cells of the innate immune system identify and engulf pathogens, which are proteolytically processed and then presented as peptides that are presented as antigens to cells of the adaptive immune system [15, 16]. But it is known that those cells can also be infected by virus suggesting that they may transport virus particles into organs and infect resident cells [17]. Increased gene expression of pro- and anti-inflammatory as well as antiviral cytokines could be observed in infected cardiac tissue [11–13, 18, 19]. But those cytokines can be expressed due to the infiltration of inflammatory cells or due to activation of resident cardiac cells triggered by viral infection [18–20].

The infection of cardiomyocytes is well documented in many publications [10, 13, 21, 22]. Here, we compare the ability of CVB3 to infect other cardiac cell types in addition to cardiomyocytes, and we investigate the virus replication in those cells.

In this study, we combined investigation in the experimentally induced viral myocarditis in mice with cell culture experiments of cardiac cells which were infected with CVB3. Therefore, we used cardiac tissue from CVB3-infected mice to evaluate the inflammatory immune response and compared these results with the inflammatory immune response in the cell culture system. Most studies focus on the virus replication in cardiomyocytes and do not consider the cardiac fibroblasts for virus replication [10, 21]. Our findings suggest that cardiac fibroblasts initiate cardiac inflammation by increased expression of proinflammatory cytokines and further aggravate viral myocarditis by higher virus replication compared to cardiomyocytes. Interestingly, macrophages as part of the innate immune system do not show high infection efficiency and no complete virus clearance was observed.

## 2. Material and Methods

### 2.1. Study Design

Male C57BL/6j (B6) mice were used at an age of 6 to 8 weeks. Animals were infected intraperitoneally with 5 × 10^5^ plaque forming units (pfu) of Coxsackievirus B3 (CVB3) (Nancy strain) diluted in PBS. As control subjects, sham infections with PBS were performed. Five animals were sham treated with PBS, whereas nine animals were infected with CVB3. Seven days later CVB3-infected B6 mice were hemodynamically characterized and compared to noninfected healthy control mice (PBS treated). This investigation conforms to the Guide for the Care and Use of Laboratory Animals published by the US NIH (NIH Publication number 85-23, revised 1996).

### 2.2. Hemodynamic Measurements

For hemodynamic measurements a microconductance catheter (1.2F) system in open-chest animals was used as described previously [23]. Animals were anesthetized (0.8–1.2 g/kg urethane and 0.05 mg/kg buprenorphine i.p.) intubated and artificially ventilated. A 1.2F-microconductance pressure catheter (SciSense, Ontario, Canada) was positioned in the left ventricle via the right carotid artery for continuous registration of pressure-volume loops. Global function was quantified by heart rate (bpm), cardiac output (*μ*L/min), stroke volume (*μ*L), stroke work (*μ*L × mmHg), and ejection fraction (%). Systolic function was assessed by maximal pressure (*P*_max⁡_ in mmHg), left ventricular contractility (*dP*/*dt*_max⁡_ in mmHg/s), and end-systolic volume (*V*_es_ in *μ*L). Diastolic function was measured by end-diastolic pressure (*P*_ed_ in mmHg), left ventricular relaxation (*dP*/*dt*_min⁡_ in mmHg/s), left ventricular relaxation time (Tau in ms), pressure halt time (PHT in ms), and end-diastolic volume (*V*_ed_ in *μ*L).

Hearts of sacrificed animals were removed and immediately frozen in liquid nitrogen and stored at −80°C for later biomolecular analyses. For validation of inflammation pieces of hearts were embedded in Tissue-Tek, sectioned, and stained with hematoxylin and eosin.

### 2.3. RNA Isolation from Tissue Sections

Frozen tissue sections were minced in Trizol and further disrupted during 10 min of vigorous shaking. To extract RNA, chloroform was added, mixed, and centrifuged. The aqueous phase containing the RNA was collected and isopropanol was added. For precipitation, the RNA solution was centrifuged 15 min at 4°C at high speed. The RNA pellet was then further purified using RNeasy Mini Kit (Qiagen) according to manufacturer's protocol. RNA concentration was determined by measuring absorbance at 260 nm using Nanodrop and stored at −80°C for further experiments.

### 2.4. Cell Culture

Murine cardiac fibroblasts were obtained by outgrowth from cardiac tissue of the left ventricle from 12-week-old male C57BL/6j mice. Therefore, the atrium as well as the right ventricle was removed using a fine surgical scissor purchased from Fine Science Tools (FST). Then, the left ventricle was cut into small pieces, which were used for the outgrowth of cardiac fibroblasts. Cells were cultured in Dulbecco's modified eagle medium (DMEM) containing 20% fetal calf serum, 100 U/mL penicillin, and 100 *μ*g/mL streptomycin (PAA). Subcultures were prepared using Trypsin/EDTA for 3 min at 37°C. Cells were characterized as fibroblasts by positive immunofluorescence staining against collagen I and negative staining for the myocyte marker desmin and the endothelial marker CD31 as described previously [24].

The cardiac muscle cell line HL-1, established from an AT-1 mouse atrial cardiomyocytes tumor lineage (kindly provided by Dr. W. C. Claycomb, Louisiana State University Medical Center, Shreveport, Louisiana), was cultured in gelatin/fibronectin precoated flasks with Claycomb medium (Sigma) containing 10% fetal calf serum (PAA), 100 *μ*M norepinephrine (Sigma), 0.3 mM ascorbic acid (Sigma), 2 mM L-glutamine, 100 U/mL penicillin, and 100 *μ*g/mL streptomycin (PAA) [25]. Subcultures were prepared as described above.

RAW264.7 cells (RAW-cells) were maintained in Dulbecco's modified eagle medium (DMEM) containing 10% fetal calf serum, 100 U/mL penicillin, and 100 *μ*g/mL streptomycin (PAA). Subcultures are prepared by scraping.

All cell culture experiments were carried out at 37°C in a humidified atmosphere with 5% CO_2_ and 95% air.

### 2.5. Virus Infection of Murine Cells

Cells were seeded in 24-well plates and grown to confluence. For starving, cells were washed once and incubated in DMEM containing 0.5% fetal calf serum, 100 U/mL penicillin, and 100 *μ*g/mL streptomycin (PAA) over night. Then, one well was used to count cell numbers to calculate the multiplicity of infection (MOI). Subsequently, starving medium was completely removed and 0.5 MOI of Coxsackievirus B3 (CVB3) (Nancy strain) diluted in DMEM without any supplements was added to infect the cells. After 60 minutes, virus was removed and cells were washed twice with PBS. Afterwards, cells were incubated in starving medium for additional 5 or 23 hours. Control cells were treated equally in the absence of virus. Cells were then lysed in RLT buffer (RNeasy Kit, Qiagen) containing *β*-mercaptoethanol as recommended in manufacturer's protocol. Total RNA was isolated using RNeasy Mini Kit (Qiagen) according to manufacturer's protocol.

### 2.6. Reverse Transcription and Relative Gene Expression Analysis

One *μ*g of total RNA isolated from cardiac tissue or 250 ng total RNA isolated from murine cells was reverse transcribed using the High Capacity Kit (Life Technologies). For reverse transcription, samples were incubated for 2 hours at 37°C followed by an inactivation step of 5 minutes at 85°C. Finally, cDNA was diluted in water to a final concentration of 10 ng/*μ*L for tissue samples and 1.25 ng/*μ*L for cell culture samples.

The relative quantification of mRNA levels was carried out on a 7900 TaqMan system (Applied Biosystems). To assess mRNA expression of target genes, real-time PCR was performed using 5 *μ*L of the gene expression master mix (Life Technologies) and 0.5 *μ*L of the gene expression assay for TNF-α (Mm00443258_m1), IL-6 (Mm00446190_m1), MCP-1 (Mm99999056_m1), IFN-*γ* (Mm00801778_m1), IFN-*β*1 (Mm00439546_s1), CAR (Mm00438361_m1), TGF-*β* (Mm00441724_m1), or IL-10 (Mm00439616_m1) purchased from Life Technologies. Each gene expression assay includes forward and reverse primers as well as the FAM-labeled probe. As template 1 *μ*L of cDNA was used in a final volume of 10 *μ*L to detect mRNA expression each performed in duplicate. Furthermore, the gene expression of CDKN1b (Mm00438167_g1) was determined and data were normalized to CDKN1b as an endogenous control using the formula 2^−ΔCt^. The expression of CDKN1b was not affected by virus infection.

CVB3 RNA was detected using the forward primer CVB3_sense (5′-CCCTGAATGCGGCTAATCC-3′), the reverse primer CVB3_antisense (5′- ATTGTCACCATAAGCAGCCA-3′) in a final concentration of 15 ng/*μ*L, and the FAM-labeled CVB3-MGB-probe (5′-TGCAGCGGAACCG-3′) in a final concentration of 0.25 pmol/*μ*L. To determine the CVB3 copy numbers, a serial dilution of a plasmid, containing the amplified CVB3 sequence, was used for the standard curve to perform the absolute gene expression analysis.

### 2.7. Quantitative RT-PCR Virus Replication

To determine the replication for the (+) or (−) RNA strand of CVB3 we used either the CVB3_antisense or CVB3_sense primer for reverse transcription, respectively. Therefore, the high capacity kit (Life Technologies) was used to transcribe 125 ng total RNA from infected cells. Instead of the random primer we used 30 ng/*μ*L of the CVB3 strand-specific primer in a final volume of 10 *μ*L. After reverse transcription at 37°C for 2 hours, followed by an inactivation step at 85°C for 5 minutes, CVB3-specific cDNA was further diluted to a final concentration of 1.25 ng/*μ*L. As described above, 1 *μ*L of these cDNAs was then used to determine the strand-specific copy numbers of CVB3. To avoid false positive results due to reverse transcription during gene expression analysis the inactivation of the RT enzyme has to be proven. Therefore, total RNA was incubated with the RT enzyme without any primer during the RT reaction followed by the inactivation step at 85°C. This control was then used for gene expression analysis for CVB3 as described and no amplification could be determined for those samples.

### 2.8. Western Blot Analysis

Cells were lysed with the lysis buffer (cell signaling) containing 1% protease as well as phosphatase inhibitors (sigma). The cell homogenates were mixed with sample buffer and separated by 12% sodium dodecyl sulfate-polyacrylamide electrophoresis gels and transferred onto a nitrocellulose blotting membrane (BioRad Laboratories). Immunoblotting was performed using specific antibodies against CAR (sc-15405, Santa Cruz) and alpha-actinin (3134, cell signaling). Secondary antibodies goat anti-rabbit IgG (Vector labartories) were used and the immunoreactive bands were visualized due to the conjugated HRP using super signal substrate (Thermo Fisher Scientific) in the chemiluminescence imaging system (FUSION, Peqlab).

### 2.9. Statistical Analysis

Continuous variables were tested for normal distribution using the Kolmogorov-Smirnov test. Since not for all data normal distribution could be proved we used Mann-Whitney *U* test to compare two groups.

For comparison of multiple groups ANOVA was used followed by Tukey's posttest. Since all data were nonnormally distributed, data were logarithmized resulting in normally distributed data before ANOVA was applied. Differences were considered statistically significant at a value of *P* < 0.05. All calculations were performed using Graph Pad Prism 5.

## 3. Results

### 3.1. Decreased LV Function after CVB3 Infection

Mice (B6) were infected intraperitoneally with 5 × 10^5^ pfu of CVB3 and hemodynamically characterized 7 days after infection. As shown in Table 1 significantly decreased LV function was determined in virus infected mice as an evidence for myocarditis despite preserved EF.

The global cardiac function is reduced in CVB3 infected animals compared to healthy controls. The hemodynamic characterization revealed significantly decreased heart rate and stroke volume resulting in impaired cardiac output. Moreover, systolic as well as diastolic function of infected animals was reduced compared to noninfected control mice suggesting that the cardiac muscle cells might be negatively affected by CVB3.

### 3.2. Cytokine Expression in Cardiac Tissue after CVB3 Infection

Gene expression of cytokines was analyzed in cardiac tissue 7 days after intraperitoneal CVB3 infection. Therefore, total RNA was isolated, reverse transcribed, and used for gene expression analysis.

**Figure 1:**
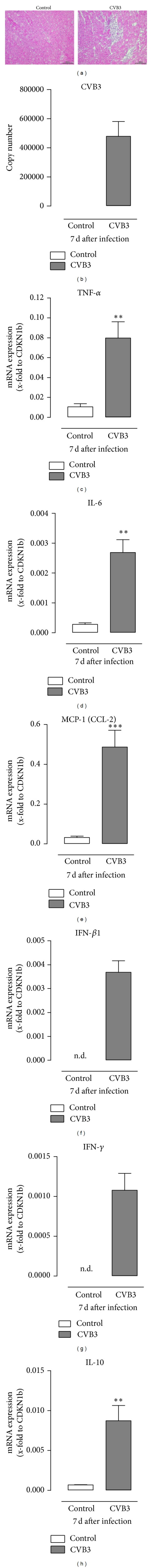
Increased cardiac inflammation in mice 7 days after CVB3 infection. (a) Hematoxylin/eosin staining of cardiac tissue from CVB3 infected B6 mice revealed a clear number of invaded inflammatory cells. (b) Virus load in cardiac tissue 7 days after CVB3 infection. ((c)–(h)) Gene expression analyses of various cytokines in cardiac tissue of healthy (white bar) and CVB3 infected (grey bar) B6 mice. Data were plotted as mean ± SEM as x-fold to the house keeping gene CDKN1b. * Significantly different versus noninfected mice (control); **P < 0.01; ***P < 0.001; n.d.: not detectable.

Moreover, about 500 000 copy numbers of CVB3 were detected per 10 ng reverse transcribed total RNA isolated from cardiac tissue of infected B6 mice (Figure 1(b)).

We further analyzed the gene expression of the cytokines TNF-α, IL-6, MCP-1, IFN-*γ*, IFN-*β*, and IL-10 in cardiac tissue. As shown in Figures 1(c)–1(e), the expression of the proinflammatory cytokines TNF-α (7-fold), IL-6 (9-fold), and MCP-1 (15-fold) was significantly increased in infected mice compared to healthy controls. As suggested, 7 days after CVB3 infection the antiviral cytokines IFN-*β* and IFN-*γ* were expressed in cardiac tissue but were not detected in healthy controls (Figures 1(f) and 1(g)). Furthermore, gene expression of the anti-inflammatory cytokine IL-10 was also significantly increased (12-fold) in the tissue of infected mice (Figure 1(h)).

### 3.3. CVB3 Leads to Severe Cell Infiltration into Cardiac Tissue

Cryosections of cardiac tissue were examined to assess the invasion of inflammatory cells into cardiac tissue of infected mice using hematoxylin and eosin-staining. As shown in Figure 1(a), cardiac tissue of infected mice demonstrated a large number of invaded inflammatory cells 7 days after infection. Different cell types in addition to the resident cardiac cells such as fibroblasts and cardiomyocytes are present in cardiac tissue. The detected increased gene expression of proinflammatory, antiviral, and anti-inflammatory cytokines in the inflamed cardiac tissue as shown in Figure 1 can originate from the entered inflammatory or from resident cardiac cells.

### 3.4. Cytokine Expression in Different Murine Cell Types after CVB3 Infection

To examine the cell specific response to virus infection, we infected murine cardiac fibroblasts, murine cardiomyocytes (HL-1 cells), and murine macrophages (RAW cells) with 0.5 MOI of CVB3. After 1 hour of infection, cells were washed and incubated for further 5 or 23 hours regarding the experimental design and time.

**Figure 2:**
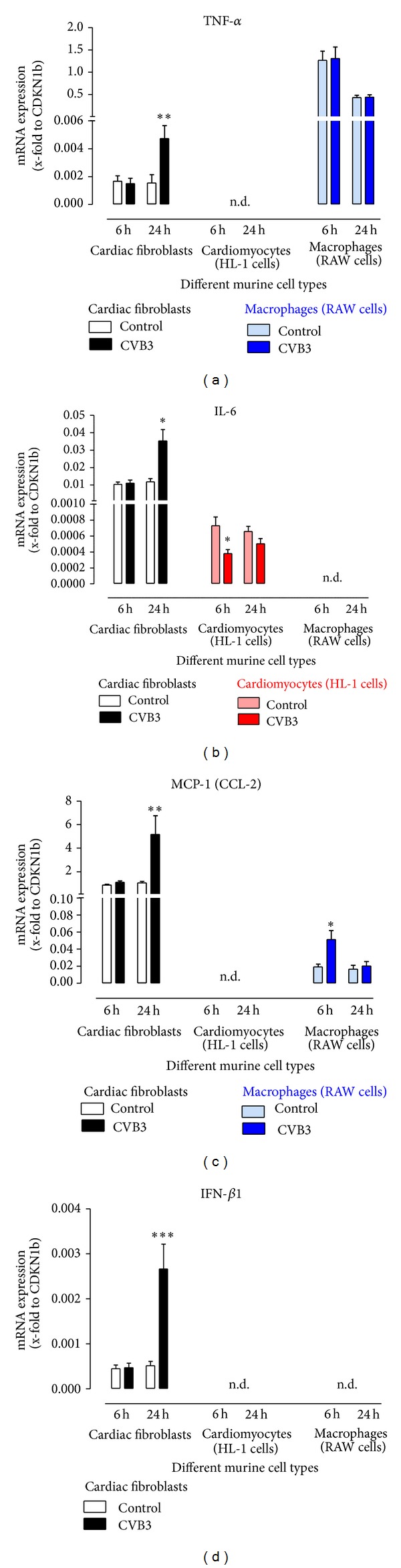
Gene expression of various cytokines after CVB3 infection of different murine cell types. Murine cardiac fibroblasts, murine cardiomyocytes (HL-1 cell line), and murine macrophages (RAW cell line) were infected with 0.5 MIO of CVB3. Gene expression of various cytokines was assessed 6 and 24 hours after CVB3 infection. To compare the absolute gene expression between the different cell types gene expression is plotted as mean ± SEM as x-fold to the house keeping gene CDKN1b.  *Significantly different versus respective noninfected cells (control); *P < 0.05; **P < 0.01; ***P < 0.001; n.d.: not detectable.

In Figure 2, the gene expression was plotted as x-fold to the house keeping gene CDKN1b to compare the absolute mRNA expression of the analyzed cytokines between the different murine cell types. The proinflammatory cytokines TNF-α, IL-6, and MCP-1 are well expressed by cardiac fibroblasts. The gene expression was increased caused by CVB3 infection (TNF-α 3-fold, IL-6 3-fold, and MCP-1 5-fold) as it was already detected in virus infected cardiac tissue. Gene expression of these three proinflammatory cytokines is hardly detectable in cardiomyocytes. Only a weak gene expression of IL-6 was measured. Furthermore, in macrophages only TNF-α was highly expressed compared to cardiac fibroblasts, but the gene expression was not altered by CVB3 infection. Nevertheless, the presence and accumulation of macrophages in inflamed cardiac tissue lead to increased TNF-α gene expression in tissue samples as shown earlier in Figure 1.

Moreover, the antiviral cytokine IFN-*γ* could not be detected in the investigated cell types. Hence, none of the here investigated cells are responsible for the virus-induced IFN-*γ* expression in cardiac tissue. In contrast, the antiviral cytokine IFN-*β* was only detectable in cardiac fibroblasts and was significantly increased 24 hours after CVB3 infection (5-fold) as already shown in the cardiac tissue of infected mice. The gene expression of the anti-inflammatory cytokine IL-10 was examined in these murine cell types. Interestingly, no expression was detectable in the here investigated cells.

### 3.5. Virus Load and Virus Replication in Different Murine Cell Types

**Figure 3:**
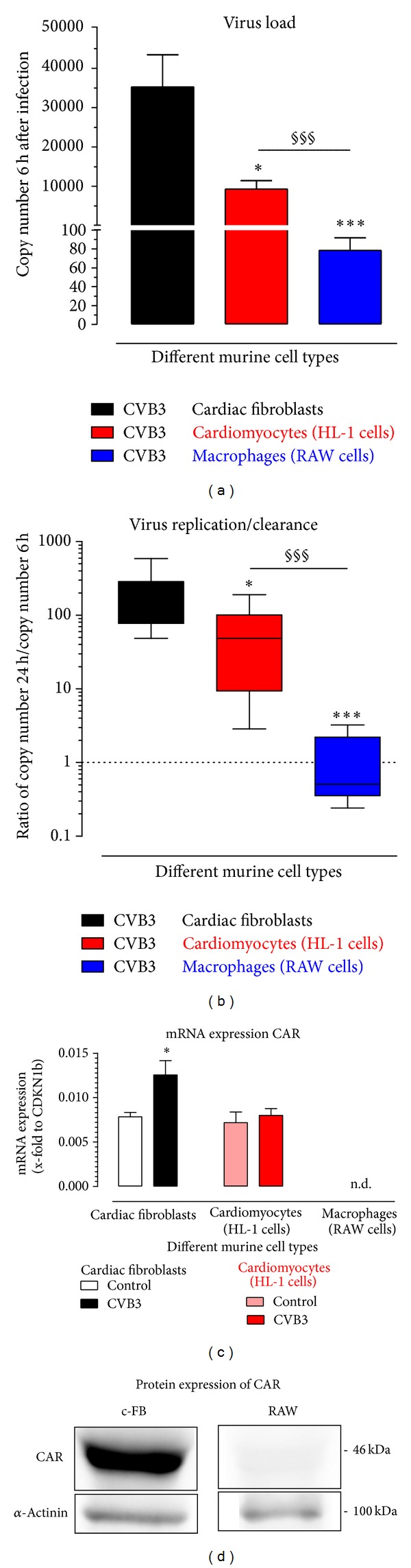
Viral infection and replication in different murine cell types. (a) CVB3 virus load in murine cardiac fibroblasts, murine cardiomyocytes, or murine macrophages was determined 6 hours after infection with 0.5 MOI CVB3. Here, cDNA transcribed with random primer was used. (b) Virus replication was determined by the ratio between detected CVB3 copy numbers 24 hours after infection and copy numbers 6 hours after infection. Copy numbers were determined using cDNA reverse transcribed with random primer. Ratios higher than one indicate virus replication. (c) Gene expression of Coxsackievirus-adenovirus receptor (CAR) was examined in cardiac fibroblasts, cardiomyocytes, and macrophages 24 hours after CVB3 infection and compared to the expression of noninfected control cells. (d) Western blot analysis revealed that CAR protein is detected in cardiac fibroblasts and is absent in macrophages (RAW cells). As endogenous control, α-actinin was performed. Data are plotted as mean ± SEM and ratios are plotted as box plots with median. ((a), (b))  *Significantly different versus cardiac fibroblasts; *P < 0.05; ***P < 0.001   ^§^significantly different versus cardiomyocytes; ^§§§^ *P* < 0.001; (c)  *significantly different versus respective noninfected control; *P < 0.05; n.d.: not detectable.

To determine the virus load in cells infected with 0.5 MOI of CVB3, total RNA was reverse transcribed using random primer and copy numbers were assessed using TaqMan analysis. As shown in Figure 3(a) murine cardiac fibroblasts reveal more than 30 000 CVB3 copies per 1.25 ng transcribed RNA 6 hours after infection. A significantly lower virus load with approximately 10 000 CVB3 copies was measured in murine cardiomyocytes. Therefore, a clear infection was determined. In contrast, in murine macrophages the detectable virus load was lower than 100 CVB3 copies 6 hours after infection. This result leads to the suggestion that cardiac fibroblasts are more efficiently infected than cardiomyocytes.

Furthermore, we determined the gene expression of one of the cellular receptors of CVB3, the Coxsackievirus-adenovirus receptor (CAR). As shown in Figure 3(c), cardiac fibroblasts revealed a similar expression of CAR as determined in cardiomyocytes. This gene expression was nearly 2 times increased in cardiac fibroblasts but not altered in cardiomyocytes 24 hours after infection. In contrast, no CAR gene expression was detectable in murine macrophages, which was confirmed by western blot analysis (Figure 3(d)).

To investigate the virus replication in different murine cell types, CVB3 copy number 24 hours after infection was normalized by the detected copy number 6 hours after infection. We plotted the ratio of CVB3 copy numbers 24 hours/6 hours. This was done to minimize the possible influence due to differences in the viral infection efficiency seen in Figure 3(a). This ratio shows the amount of viral replications between 6 and 24 hours. As shown in Figure 3(b), in cardiac fibroblasts CVB3 copy number was nearly 200 times increased after 24 hours compared to the determined copy number 6 hours after infection. In cardiomyocytes CVB3 copy numbers after 24 hours were 65-fold increased compared to CVB3 copy numbers after 6 hours.

In macrophages nearly the same amount of CVB3 copies was determined after 24 and after 6 hours. Thus, cardiac fibroblasts as well as cardiomyocytes replicate CVB3 in contrast to macrophages, where no changes could be observed. According to this result, neither virus replication nor virus clearance was determined in macrophages.

Furthermore, the amount of isolated total RNA from macrophages does not differ between samples with or without virus, suggesting that no cell lysis due to the cytolytic cycle of the virus has occurred within 24 hours after infection (Figure 5(d)). Therefore, it can be suggested that the macrophages are not killed by CVB3 within 24 hours. Interestingly, a decreasing amount of isolated total RNA was detected with increasing MOI of CVB3 in cardiac fibroblasts after 24 hours. Furthermore, decreased amounts of total RNA were also detected in infected HL-1 cells. Those results suggest that in the culture of cardiac fibroblasts as well as cardiomyocytes cell lysis occurs due to the cytolytic cycle of the virus within 24 hours after infection.

### 3.6. Copy Numbers of Viral Genome and Intermediate Strand for Virus Replication

Since it is not clear if macrophages are really infected by CVB3, additional experiments were performed. The detected CVB3 copies can also correspond to incompletely removed virus particles instead of virus RNA within the cells due to a reliable virus infection. The intermediate strand, which is necessary for virus replication, can only be detected in infected cells and cannot correspond to the incompletely removed virus particles.

Therefore, the total RNA isolated from infected cells was used to identify the copy number of the (+) strand which represents the number of RNA virus genome of CVB3 and the (−) strand which is synthesized in infected cells as a template to produce the (+) strand for new virus particles. Therefore, the (−) strand is an intermediate for virus replication. As shown earlier in Figure 3, macrophages were hardly infected by CVB3 which is further strengthened by the fact that no gene expression of CAR, known as CVB3 receptor, was determined in macrophages.

**Figure 4:**
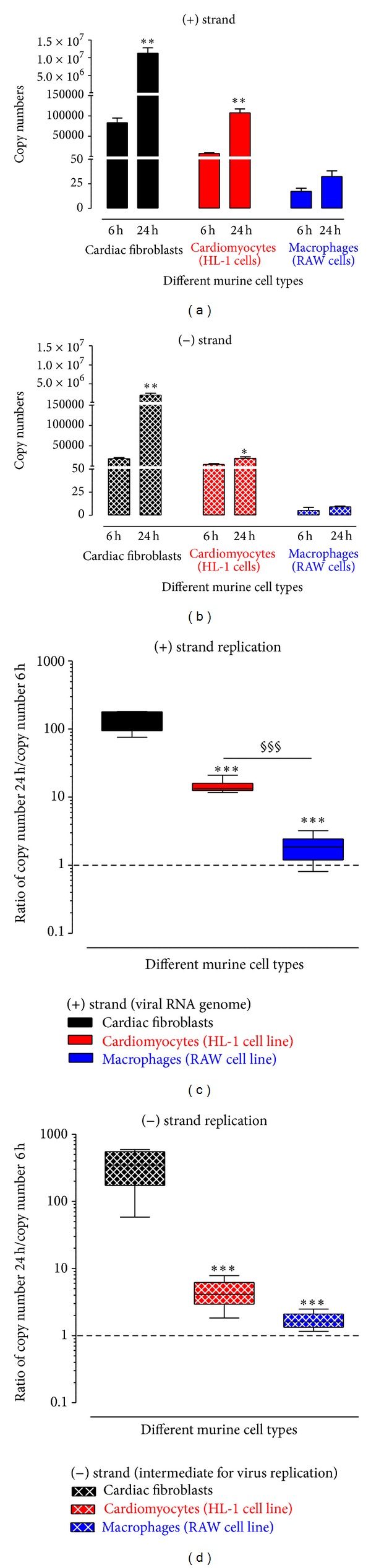
CVB3 copy numbers of the viral RNA genome (+) strand as well as the intermediate (−) RNA strand for virus replication. The copy number of the viral RNA genome (+) strand and the copy number of the intermediate (−) strand needed for virus replication were determined using strand-specific primers for reverse transcription followed by TaqMan analysis. (a) Copy numbers of (+) strand (viral RNA genome) of CVB3 6 hours and 24 hours after infection of murine cardiac fibroblasts, murine cardiomyocytes (HL-1 cell line), and murine macrophages (RAW cell line). (b) Copy numbers of (−) strand (intermediate RNA strand for virus replication) of CVB3 6 hours and 24 hours after infection of murine cardiac fibroblasts, murine cardiomyocytes (HL-1 cell line), and murine macrophages (RAW cell line). ((c), (d)) Ratio between copy numbers 24 hours compared to copy numbers 6 hours after CVB3 infection for (c) (+) strand and (d) (−) strand. Ratios higher than one demonstrate virus replication. ((a), (b))  *Significantly different versus copy numbers 6 hours after infection; *P < 0.05; **P < 0.01. Data are plotted as mean ± SEM. ((c), (d))  *Significantly different versus cardiac fibroblasts; ***P < 0.001 ^§^significantly different versus cardiomyocytes; ^§§§^ *P* < 0.001. Ratios are plotted as box plots with median.

Therefore, CVB3_antisense primer was used as a CVB3 (+) strand-specific primer to transcribe this specific RNA strand into cDNA (+). Accordingly, CVB3_sense primer was used as a specific primer to transcribe the CVB3 (−) strand into cDNA (−). To avoid false positive results the enzyme reverse transcriptase has to be inactivated prior to the gene expression analysis [26]. After inactivation both types of cDNA were used for TaqMan analysis to evaluate the specific copy numbers for each strand separately. As shown in Figures 4(a) and 4(b), in cardiac fibroblasts (black bars) as well as in cardiomyocytes (red bars) the copy number of both strands was significantly increased 24 hours after infection compared to copy numbers 6 hours after infection. Contrarily, in macrophages the copy numbers do not differ remarkably between 6 hours and 24 hours after infection.

Since macrophages display less than 100 copies, it is unclear if these detected CVB3 copies correspond rather to the incompletely removed virus particles than to those virus particles which have infected macrophages. An evidence for infection would be a detectable expression of the intermediate (−) strand of CVB3. As shown in Figure 4(b), the replication of CVB3 was proven by detection of the (−) strand of CVB3 in all three cell types. Accordingly, macrophages were infected by CVB3 even if no gene expression of CAR was detectable.

To compare the replication of each CVB3 RNA strand in the different cell types, in Figures 4(c) and 4(d) we plotted the ratio of CVB3 copy numbers 24 hours/6 hours. This was done to minimize the possible influence due to differences in the viral infection efficiency seen in Figure 4(a). Then, viral replication between 6 and 24 hours can clearly be identified. In cardiac fibroblasts the (+) and the (−) strands of CVB3 were dramatically increased between 6 and 24 hours ((+) strand > 100-fold, (−) strand > 300-fold). This is significantly different from the detected ratio in cardiomyocytes and macrophages. In cardiomyocytes the replication of the intermediate CVB3 (−) strand is only slightly increased (4-fold) but is enough to clearly replicate the CVB3 (+) strand (14-fold) between 6 and 24 hours. In macrophages, only a very weak replication was detectable for the (+) strand (1.7-fold) and the (−) strand (1.8-fold).

### 3.7. Macrophages Cannot Clear Virus Completely

**Figure 5:**
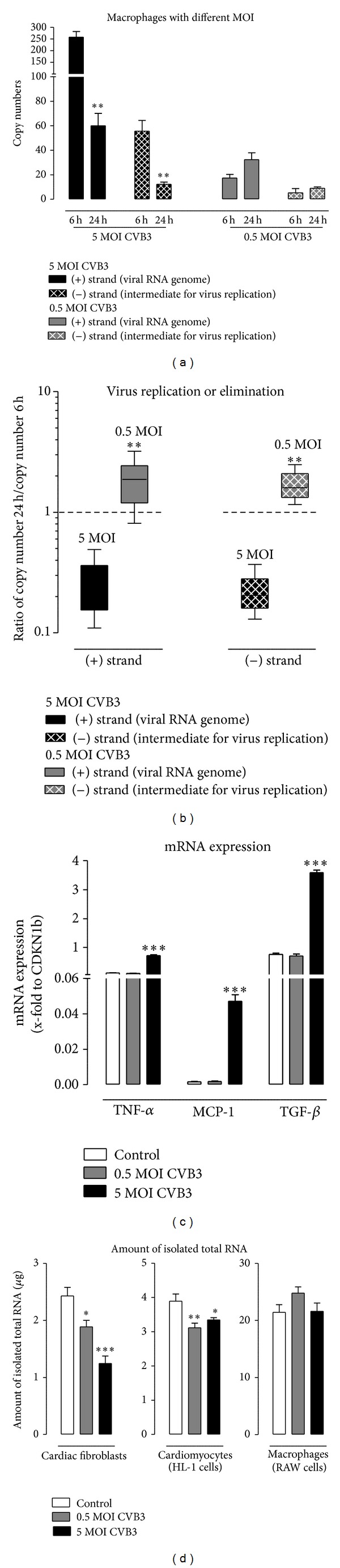
Virus clearance or persistence in macrophages infected with high or low MOI of CVB3. Murine macrophages (RAW cells) were infected with 5 MOI or 0.5 MOI of CVB3. The copy number of the viral RNA genome (+) strand and the copy number of the intermediate (−) strand needed for virus replication were determined using specific primers for reverse transcription followed by TaqMan analysis. (a) Copy numbers of both CVB3 strands using different MOI 6 hours and 24 hours after infection. (b) Ratio between copy numbers after 24 hours after infection compared to 6 hours after CVB3 infection. Ratios lower than 1 show virus elimination. (c) To determine gene expression of macrophages infected with 5 or 0.5 MOI of CVB3 total RNA was reverse transcribed using random primer and gene expression of TNF-α, MCP-1, and TGF-*β* was determined 6 hours after infection. (d) 24 hours after infection with CVB3 cells were washed to remove cell debris and then total RNA was isolated from the remaining cells. The isolated amount of total RNA is plotted and revealed a decrease due to virus infection followed by cell lysis in cardiac fibroblasts and cardiomyocytes but not in macrophages. (a)  *Significantly different versus copy numbers 6 hours after infection; **P < 0.01. Data are plotted as mean ± SEM. (b)  *Significantly different versus 5 MOI; **P < 0.01. Ratios are plotted as box plots with median. (c)  *Significantly different versus noninfected control, *P < 0.05; **P < 0.01; ***P < 0.001. Data are plotted as mean ± SEM.

Since macrophages belong to the innate immune system virus elimination was expected. All previous experiments were carried out with a 0.5 MOI of CVB3. In Figure 5, macrophages were also infected with 5 MOI of CVB3 and again the isolated total RNA was reverse transcribed with CVB3 (+) and (−) strand specific primers. These resulted cDNAs were further used for TaqMan analysis to determine the strand specific copy numbers.

After infection with 5 MOI of CVB3, macrophages exhibit higher copy numbers 6 hours after infection for (+) and (−) strands compared to macrophages infected with 0.5 MOI of CVB3 as shown in Figure 5(a). 24 hours after CVB3 infection with 5 MOI the copy numbers for (+) as well as for (−) strands are significantly decreased ((+) strand −3.6-fold, (−) strand −4.5-fold) nearly to the level of CVB3 copy numbers detected 24 hours after infection with 0.5 MOI, whereas no significant changes were determined in macrophages infected with 0.5 MOI of CVB3 between 6 hours and 24 hours after infection.

To determine if virus replication or virus clearance occurs in macrophages after CVB3 infection, the ratio of CVB3 copy numbers 24 hours/6 hours is plotted in Figure 5(b). It is clearly shown for the (+) and for the (−) strands that macrophages are able to eliminate CVB3 at 5 MOI, but no clear virus replication or elimination is documented for 0.5 MOI of CVB3.

Furthermore, the gene expression of the proinflammatory cytokine TNF-α is increased (6-fold) in macrophages infected with 5 MOI of CVB3 6 hours after infection, in contrast to macrophages infected with 0.5 MOI, in which no alteration was measured. Similar results were found for the chemokine MCP-1. The gene expression remains unchanged after infection with 0.5 MOI of CVB3 but increases after infection with 5 MOI (28-fold). However, expression of the profibrotic and anti-inflammatory cytokine TGF-*β* is increased by 5 MOI CVB3 (4.7-fold) but remains unchanged after CVB3 infection with 0.5 MOI.

## 4. Discussion

In this study, we elucidate a cell type specific immune response to CVB3 using murine cardiac fibroblasts, cardiomyocytes, and macrophages. Additionally, we document differences in virus infection, virus replication, or elimination between these cell types. Cardiac fibroblasts are infected with high efficiency by CVB3 and resulted in a much higher virus replication compared to cardiomyocytes. Interestingly, macrophages were infected by CVB3 with a very low efficiency but revealed virus degradation only when infected with high virus concentrations. However, no complete virus clearance by macrophages was observed.

### 4.1. Inflammation during Viral Myocarditis

First, we analyzed the cardiac tissue from B6 mice, which were peritoneally infected with CVB3, or control animals, which were PBS treated. Seven days after infection, the cardiac tissue from infected animals clearly exposes inflammatory cellular infiltrates, which conforms to the general definition of myocarditis [1, 3, 6]. Moreover, the cardiac tissue of infected animals clearly exhibits copy numbers of CVB3.

Viral myocarditis can be divided into 3 phases. The first acute phase is when susceptibility to viral infection is determined and direct consequences of virus-induced cytotoxicity in the absence of inflammatory cells can be seen within the first 3 days after infection [3, 18]. The next phase, the subacute phase, is defined by active replication of virus occurring in the myocardium (days 4–14) and the first wave of infiltrating cells [18]. Finally, during the chronic phase of viral myocarditis virus replication has ceased but viral genome persists in the myocardium. Furthermore, remodeling takes place after myocardial damage due to virus infection and inflammation during phases 1 and 2 [3, 18]. Our data, 7 days after CVB3 infection, are consistent with the subacute phase of viral myocarditis.

Inflammatory cells already infiltrated the cardiac tissue and various cytokines, including proinflammatory and antiviral cytokines, are expressed. In this study, we showed increased expression levels of proinflammatory cytokines (TNF-α, IL-6, and MCP-1) as well as antiviral cytokines (IFN-*β* and IFN-*γ*) and an anti-inflammatory cytokine (IL-10) due to viral myocarditis in cardiac tissue. Those cytokines play an important role to regulate the increasing inflammation due to virus infection. In transgenic mice overexpressing IL-6 in cardiac tissue, an association with elevated basal inflammation was shown [27]. Interestingly, anti-inflammatory treatments with a soluble TNF receptor or with a neutralizing antibody against chemokines can reduce cardiac inflammation and prevent cardiac damage in experimental models [11, 28] but not in randomized clinical trials [29]. On the other hand, antiviral treatment using IFN-*β* or IFN-α improves cardiac function in patients with persistent virus infection [30, 31] which is in line with the finding that an IFN-*β* neutralizing antibody increased virus replication in cardiomyocytes [10].

Those proinflammatory or antiviral cytokines are present in the cardiac tissue and it is not clear which cell type is mainly responsible for a specific cytokine elevation. Here, we infected three different cell types in vitro with 0.5 MOI of CVB3 and analyzed the cell type specific cytokine expression. Therefore, we used murine primary cardiac fibroblasts, the murine cardiomyocyte cell line HL-1, and the murine macrophage cell line RAW. Fibroblasts are found throughout the cardiac tissue surrounding cardiomyocytes, and they present the most numerous cell types in the heart. Macrophages constitute a major part of the innate immune system. Hence, they are at the front line of defense against fungi, bacteria, and viruses [15]. Interestingly, macrophages reside in the healthy cardiac tissue, although they are not as frequent as cardiomyocytes or fibroblasts. But during diseases their number is increased [16].

The expression of proinflammatory cytokines (TNF-α, IL-6, and MCP-1) was increased in cardiac fibroblasts due to CVB3 infection. In cardiomyocytes a very weak expression of IL-6 and no detectable expression of TNF-α and MCP-1 were determined. Interestingly, the expression of IL-6 was decreased in cardiomyocytes 6 hours after viral infection. Macrophages express TNF-α and MCP-1, whereas MCP-1 expression was slightly increased due to CVB3 infection and TNF-α expression remains unchanged. If macrophages were infected with a 10-fold higher amount of CVB3 (5 MOI) gene expression of the proinflammatory cytokines TNF-α and MCP-1 are then upregulated after 6 hours. Moreover, the chemokine MCP-1 is expressed nearly 100-fold higher in cardiac fibroblasts than in macrophages but virus leads to increased expression of MCP-1 in both cell types.

The antiviral cytokine IFN-*γ* and anti-inflammatory cytokine IL-10 are mainly related to T-cells. No expression of IFN-*γ* and IL-10 was detected in the here investigated cell types [32], whereas IFN-*β* is well expressed in cardiac fibroblasts. The expression of IFN-*β* is related to Toll-like receptors, which are known to recognize nucleic acids such as viral RNA. This mechanism also belongs to the innate immune system. It was recently shown that high IFN-*β* expression levels diminished virus replication in cardiomyocytes [10].

### 4.2. Virus Infection and Replication after CVB3 Infection

The coxsackievirus and adenovirus receptor (CAR) was identified as the cellular receptor for CVB3 [33–35]. Murine cardiac fibroblasts, cardiomyocytes, and macrophages were tested for the gene expression of CAR. The cardiac fibroblasts revealed a high expression of CAR similar to cardiomyocytes, which was further increased during 24 hours after virus infection. In cardiomyocytes, CAR expression remains at the same level. Furthermore, it is reported that CVB3 infected cardiomyocytes revealed a downregulation of CAR within 3 passages [21]. For murine macrophages no gene expression of CAR could be determined but very low infection by CVB3 was still detectable. Further no CAR protein was detected in macrophages. This suggests that CAR is not expressed or lower expressed than the detection level, which would be consistent with the determined low infection. Another reason might also be that CVB3 particles enter the cells after attaching to another receptor, which is also reported as CVB3 receptor, the decay-accelerating factor (DAF)/CD55 [36]. In another study on human cardiac cells an internalization of CVB3 is reported in the presence of CD55 and CAR-blocking monoclonal antibodies. Furthermore, they found using immunoprecipitation that CVB3 binds at least five additional cellular receptors, distinct from CAR and CD55, on the surface of human cardiac cells [37]. This suggests that CVB3 appears to employ multiple receptors to internalize in cardiac cells.

After entering the cells, virus typically starts replication [38, 39]. In general, virus replication consists of two steps. First, a complementary negative strand from virion RNA is synthesized, which then serves as a template for the synthesis of a large amount of progeny positive-strand RNAs [40, 41]. Since the genome of CVB3 consists of a (+) strand RNA, a (−) strand RNA has to be synthesized as an intermediate for virus replication. Murine cardiac fibroblasts as well as cardiomyocytes reveal a severe infection of CVB3 in contrast to macrophages, in which less than 100 copies were detected. It was unclear if these detected CVB3 copies correspond to incompletely removed virus particles or to virus particles within the cells due to a reliable virus infection. Here, the evidence, that the macrophages are infected by virus, is provided by the detection of the intermediate (−) strand of CVB3 [40].

**Figure 6:**
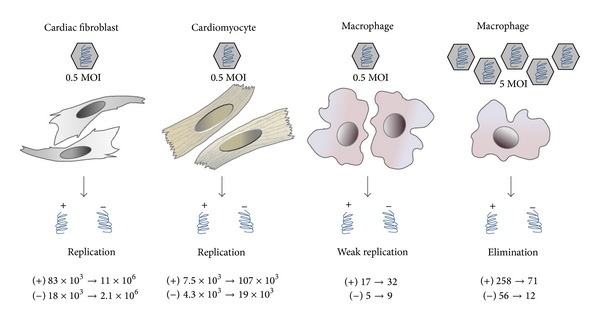
Comparison of CVB3 replication in different cell types. Due to viral infection cardiac fibroblasts revealed 132-fold increase (83 × 10^3^→ 11 × 10^6^ copies) of the viral (+) RNA strand between 6 and 24 hours leading to a severe virus replication, whereas in cardiomyocytes 14-fold increase (7.5 × 10^3^→ 107 × 10^3^ copies) of the viral (+) RNA strand was measured. In macrophages, low virus concentration leads to weak virus replication (17 → 32 copies). High virus concentration leads to virus elimination but not to complete virus clearance (258 → 71 copies). The copy numbers of both CVB3 strands 6 hours and 24 hours after infection are given (6 h → 24 h).

Furthermore, cardiac fibroblasts reveal a tremendous virus replication for the progeny (+) strand as well as for the intermediate (−) strand. In fibroblasts both strands were highly amplified between 6 hours and 24 hours after CVB3 infection as seen in Figure 6. The (+) strand was 132-fold increased between 6 and 24 hours (83 × 10^3^→ 11 × 10^6^ copies). In contrast, in cardiomyocytes less CVB3 copy numbers were detected and the amount of the intermediate (−) strands was hardly increased between 6 hours and 24 hours after infection. But this amount was obviously sufficient to produce an increased amount of the progeny (+) strand RNA leading to a 14-fold increase of the (+) strand (7.5 × 10^3^→ 107 × 10^3^ copies).

Since macrophages are part of the innate immune system they are often the first to identify and engulf invading pathogens [15]. Furthermore, they are responsible for antigen presentation for T-cells. A similar function is also reported for dendritic cells. They identify, engulf pathogens, and finally present their parts as antigens [17]. Here, we used the macrophage cell line RAW264.7. An infection with CVB3 was proven for this specific cell type, since the positive as well as the negative RNA strand of CVB3 was determined. Interestingly, Weinzierl et al. recently documented this in dendritic cells [17]. There, CD11c^+^ dendritic cells derived from C57BL/6 were able to completely eliminate the negative RNA strand of CVB3 within 8 days after infection. In our cell culture experiments, macrophages infected with 5 MOI reduce the number of (+) as well as (−) strands but no alteration within 24 hours was measured in macrophages infected with 0.5 MOI. This might be an explanation for virus persistence in the cardiac tissue, since macrophages can only reduce, but not completely clear, the infected tissue from CVB3.

In summary, cardiac fibroblasts as well as cardiomyocytes are target cells for CVB3 infection. Both cardiac cell types replicate the intermediate (−) strand and the progeny (+) strand RNA whereas no reliable increasing virus RNA was detected in macrophages. At higher virus concentrations, macrophages eliminate CVB3 but no complete virus clearance could be observed suggesting a persistent viral infection. Cardiac fibroblasts aggravate CVB3-induced viral myocarditis by exacerbated virus replication compared to cardiomyocytes. On the other hand, they initiate cardiac inflammation by upregulating the gene expression of proinflammatory cytokines such as TNF-α, IL-6, and MCP-1 as well as antiviral IFN-*β* which was not observed in cardiomyocytes infected by CVB3. Future studies have to elucidate whether these in vitro changes using different cell types are important in vivo investigating murine models as well as human patients with myocarditis.

In conclusion, we suggest that cardiac fibroblasts play an important part in the pathology of CVB3-induced myocarditis. Those results should trigger further research aiming cardiac fibroblasts as another important contributor of virus replication which can exacerbate myocarditis.

## Figures and Tables

**Table 1:** Hemodynamic function of B6 mice 7 days after CVB3 infection.

	B6-PBS treated	B6-CVB3 treated	*P* value
Global function			
Heart rate [bpm]	595 ± 13	549 ± 16	0.042
Cardiac output [*μ*L/min]	26041 ± 2297	14838 ± 1669	0.004
Stroke volume [*μ*L]	44 ± 4	27 ± 3	0.012
Stroke work [*μ*L*·*mmHg]	3468 ± 440	1697 ± 244	0.007
Ejection fraction [%]	74 ± 3	67 ± 5	0.298
Systolic function			
*P*_max⁡_ [mmHg]	84 ± 5	69 ± 3	0.029
dP/dt_max_ [mmHg/s]	9147 ± 893	6182 ± 663	0.042
*V*_es_ [*μ*L]	18 ± 3	14 ± 2	0.363
Diastolic function			
*P*_ed_ [mmHg]	3.4 ± 0.5	2.3 ± 0.3	0.083
dP/dt_min_ [mmHg/s]	−5024 ± 451	−3565 ± 300	0.019
Tau [ms]	9.4 ± 0.2	11.8 ± 0.5	0.001
PHT [ms]	5.2 ± 0.2	7.0 ± 0.5	0.007
*V*_ed_ [*μ*L]	60 ± 6	41 ± 3	0.019
